# Post-Transjugular Intrahepatic Portosystemic Shunt (TIPS) Hepatic Encephalopathy—A Review of the Past Decade’s Literature Focusing on Incidence, Risk Factors, and Prophylaxis

**DOI:** 10.3390/jcm13010014

**Published:** 2023-12-19

**Authors:** Karina Holm Friis, Karen Louise Thomsen, Wim Laleman, Sara Montagnese, Hendrik Vilstrup, Mette Munk Lauridsen

**Affiliations:** 1 Department of Gastroenterology and Hepatology, University Hospital of Southern Denmark, Finsensgade 35, 6700 Esbjerg, Denmark; 2 Department of Hepatology and Gastroenterology, Aarhus University Hospital, 8200 Aarhus, Denmark; 3 Department of Gastroenterology and Hepatology, University Hospitals Leuven, KU Leuven, 3000 Leuven, Belgium; 4 Department of Medicine, University of Padova, 35122 Padova, Italy

**Keywords:** liver cirrhosis, portal hypertension, variceal bleeding, cognition, brain dysfunction, ascites, time trend

## Abstract

Transjugular intrahepatic portosystemic shunt (TIPS) is an established treatment for portal hypertension and its’ complications in liver cirrhosis, yet the development of hepatic encephalopathy (HE) remains a significant concern. This review covers the reported incidence, risk factors, and management strategies for post-TIPS HE over the past decade. Incidence varies widely (7–61%), with factors like age, liver function, hyponatremia, and spontaneous portosystemic shunts influencing risk. Procedural aspects, including TIPS timing, indication, and stent characteristics, also contribute. Pharmacological prophylaxis with lactulose and rifaximin shows promise, but current evidence is inconclusive. Procedural preventive measures, such as shunt embolization and monitoring portal pressure gradients, are explored. Treatment involves pharmacological options like lactulose and rifaximin, and procedural interventions like stent diameter reduction. Ongoing studies on novel predictive markers and emerging treatments, such as faecal microbiota transplant, reflect the evolving landscape in post-TIPS HE management. This concise review provides clinicians with insights into the multifaceted nature of post-TIPS HE, aiding in improved risk assessment, prophylaxis, and management for patients undergoing TIPS procedures.

## 1. Introduction

Transjugular intrahepatic portosystemic shunt (TIPS) is an effective treatment for patients with liver cirrhosis and portal hypertension with intractable ascites and variceal bleeding and has been used since 1988 [1]. The indications for TIPS placement have been expanded and refined in recent years. Preemptive TIPS is now the new standard in many centres, and indications are broadened to include non-cirrhotic portal hypertension, portal vein thrombosis, Budd–Chiari syndrome, hepatorenal and hepatopulmonal syndrome, and cases of hepatocellular carcinoma with portosystemic symptoms [2,3,4,5,6,7,8,9]. Although technical advancements have decreased post-TIPS morbidity significantly, hepatic encephalopathy (HE) remains a well-known and debilitating complication of TIPS placement (HE) even when present as minimal or grade I HE (‘covert HE’) [10,11,12,13]. Post-TIPS HE severely detracts from the utility of TIPS by further reducing the recipients’ quality of life and being a leading cause of admissions and, as such, an economic burden to healthcare systems. Therefore, post-TIPS HE is an essential point of attention to any clinician caring for patients with liver cirrhosis [14,15,16]. This article gives an overview of the reported incidence, prevalence, and pathogenesis of post-TIPS HE within the past decade and discusses risk factors and predictors as well as prophylaxis and treatment.

## 2. Literature Search

**Figure 1:**
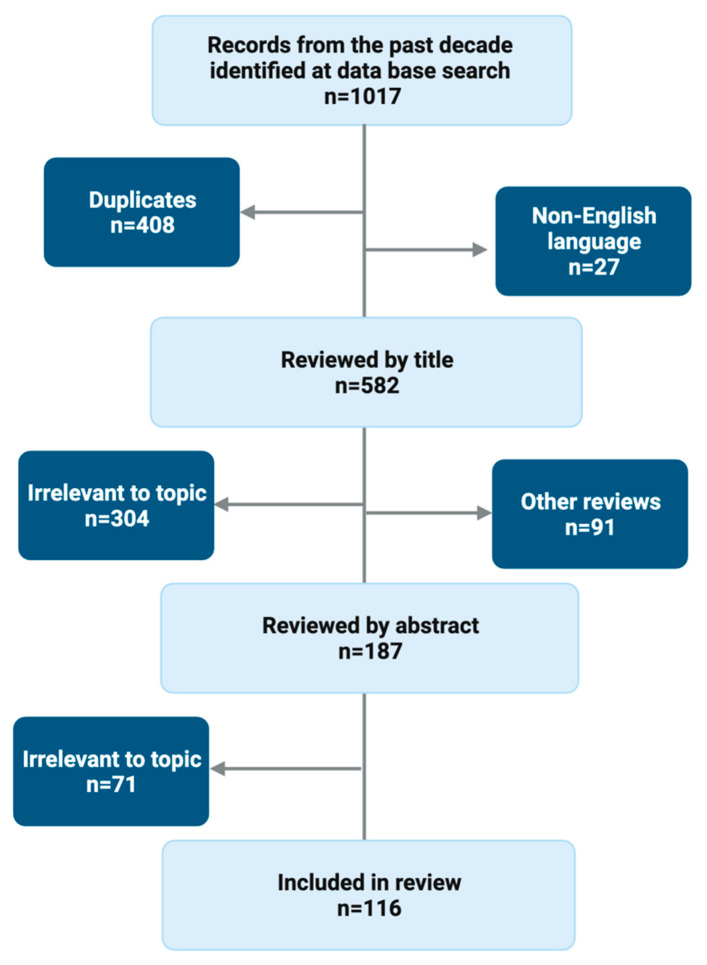
Literature search flowchart.

We searched databases for literature going ten years back using the search terms ‘TIPS AND hepatic encephalopathy’ and ‘TIPS AND hepatic encephalopathy + [title of subheading]’. We applied both the whole term ‘trans-jugular portosystemic shunt’ and the abbreviations ‘TIPSS’ and ‘TIPS’. We chose ten years because we wanted to analyse post-TIPS HE in the clinical routine setting. There were 582 non-duplicate, English-language publications, and all were screened for relevance to the sub-headings presented below (Figure 1). We found 375 original manuscripts to be irrelevant after screening first the title and secondly the abstract. Another 114 were other review articles, and these were also excluded. One hundred and sixteen manuscripts were included in this review. Fourteen articles served as background literature. 

## 3. Incidence

**Figure 2:**
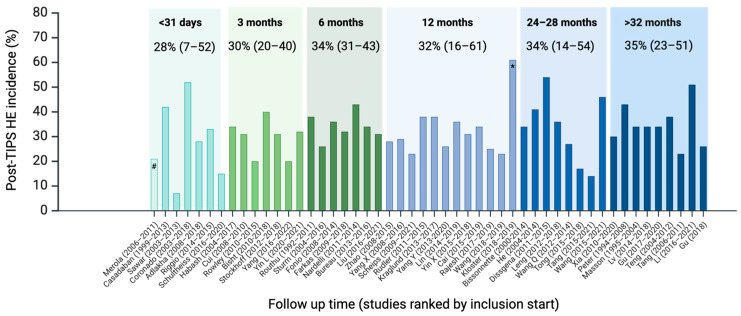
Fifty studies published within the past decade reporting post-TIPS HE absolute risk at a given follow-up time, not including studies in non-cirrhosis cohorts and HCC. Studies are grouped according to mean follow-up time and within each time category ordered after the year in which recruitment began. Numbers give mean post-TIPS HE absolute risk and range in brackets. The studies are too few to conduct meaningful statistical calculations. Still, the mean post-TIPS HE risk is not significantly different between studies that started inclusion before and after 2013 or before or after 2016 (*p* = 0.8 and 0.9). # Only ascites indication, one week follow-up; * Viatorr controlled expansion stent 8 or 10 mm, *n* = 33.

HE is a common complication after TIPS placement, but the reported incidence varies considerably among cohorts and the calendar time of recruitment (Figure 2). Studies published within the past decade, including patients from 2004 to 2022, report post-TIPS HE incidence ranging from 7% to 61%. This large variance is present regardless of the follow-up time as illustrated by 1, 3, and 6-month cumulative incidences from 7% to 52%, 20% to 40%, and 26% to 43%; and 12, 24–28, and >32 month incidences from 23% to 61%, 17% to 54%, and 26% to 51%. 

Despite recent clinical and procedural advancements, there has been no overall trend towards a decrease in incidence over the years, as illustrated by Figure 2, where studies are ordered according to the year when patient recruitment began (Figure 2) [17,18,19,20,21,22,23,24,25,26]. The studies are too few to conduct meaningful statistical calculations. Still, the mean post-TIPS HE risk is not significantly different between studies that started inclusion before and after 2013 or before or after 2016 (*p* = 0.8 and 0.9). The highly varying incidence among the studies can be ascribed to patients’ individual risk factors, local selection and patient optimisation regimen, use of pre-TIPS HE prophylaxis, the stent type and diameter, and the TIPS procedure volume at each site. The significance of such clinical or modifiable factors is illustrated in a large cohort including >700 patients where the incidence was 15.5% but as much as 65% in the subgroup with Child-Pugh C cirrhosis, and only 6.4% in Child-Pugh B patients with a small stent diameter [27]. 

Another difficulty in describing post-TIPS HE incidence is dissimilar reporting strategies, even between similar centres and cohorts. Also, the statistical data treatment may be suboptimal. For example, many studies report incidence without considering competing events, such as death, leading to deflated risk estimates [28]. 

Incidence reports also differ with differing diagnostic and reporting strategies on minimal HE (MHE) (cf. below). 

The time course of post-TIPS HE is defined by a steep rise in HE incidence in the first month after TIPS insertion, where liver function often takes a post-procedural hit [29]. The risk of new-onset episodic or recurrent HE remains high during the first year after TIPS but with the well-known post-TIPS improvement in muscle mass and fewer stressful events, the risk seemingly stagnates or declines [29,30,31,32,33,34]. Most cases of post-TIPS HE respond to lactulose and rifaximin treatment, but it is noteworthy that persistent post-TIPS HE is reported in 4–10% [20,35,36,37,38].

## 4. Pathogenesis

The pathogenesis of post-TIPS HE is no different from pre-TIPS HE, but TIPS placement induces an abrupt change in the splanchnic and intrahepatic hemodynamics and a systemic gut-derived toxin load [39]. TIPS compromises the functional architecture of the liver, including the mechanisms for removing ammonia, viz., glutamine and urea synthesis. Ammonia crosses the blood–brain barrier (BBB) in parallel with blood concentration. The resulting astrocytic glutamine synthesis acts as an osmotic load with astrocyte swelling and dysfunction [40,41]. Secondary brain metabolic consequences include increased neuronal inhibitory transmission by higher GABA-ergic tone. Systemic low-grade inflammation activation damages the integrity of the BBB, facilitating ammonia efflux and neuroinflammation [40]. Hepatic macrophage activation is not appeased with TIPS and may continue contributing to systemic and cerebral inflammation activation after TIPS [42]. Cirrhotic gut dysbiosis likely plays a pathogenic role, but the issue and the related therapeutic potential are not yet fully elucidated. Bile acids and, in particular, one of their receptors (FXR receptor), seem to play a role in HE development, and a recent preprint applying untargeted portal vein metabolomics pre and post-TIPS even suggests that certain conjugated bile acids may exert a protective role in post-TIPS HE [43,44]. 

The clinical manifestations of post-TIPS HE range from mild cognitive impairment to confusion and, ultimately, coma. It is traditionally graded after severity according to the West Haven Criteria, ranging from MHE through grade I-IV HE [45]. MHE is not clinically detectable but can only be diagnosed through neuropsychological or electrophysiological testing [46]. Post-TIPS has no clinical traits distinguishing it from other cases of HE. 

## 5. The Burden of Post-TIPS HE

The 30-day readmission rate after TIPS placement may be as high as 30%, with at least 1/3 being HE-related. The average cost of pr. readmission was USD 45,751 [47]. HE of any grade, pre- or post-TIPS, is associated with a decreased quality of life and, in cases requiring admission, a complete loss of autonomy. MHE often results in driving difficulties and an increased tendency to fall and, as such, lower self-sufficiency [48,49]. In line with that, after experiencing HE, patients report fear of recurrence, multiple losses, dependence on others, and social isolation [50]. HE of any grade further impacts the lives of caregivers [51,52,53]. No evidence supports that post-TIPS HE is more deadly than HE occurring independently of TIPS placement where 1-year mortality after an HE episode is reported to be as high as 65% [14]. 

## 6. Risk Factors

**Figure 3:**
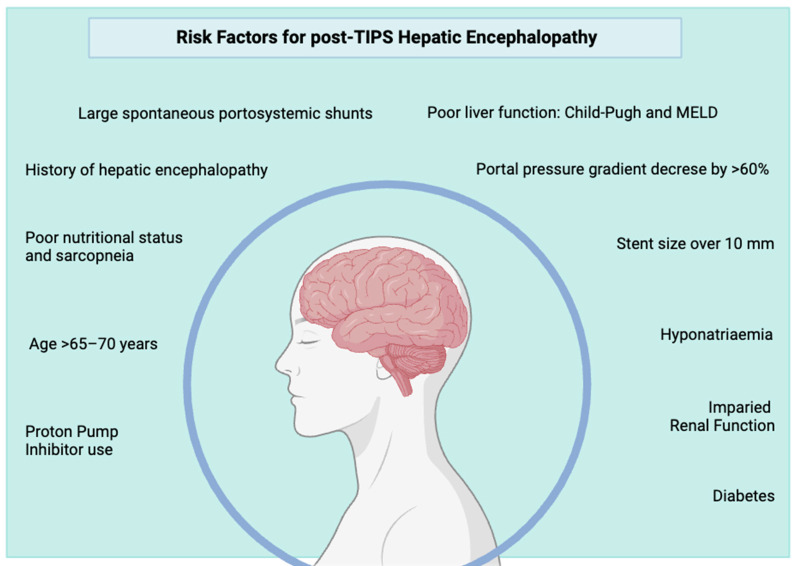
Overview of the most well-documented risk factors for post-TIPS hepatic encephalopathy. Many are modifiable and should be addressed before elective TIPS.

The occurrence of post-TIPS HE varies significantly from patient to patient, mainly depending on individual risk factors. Some of these are given, while others can be mitigated. Identifying individual risk factors is always important and aids in selecting patients most likely to benefit from the procedure, identifying those needing closer follow-up and monitoring and considering preventive anti-HE treatment. All the known precipitating factors for OHE represent risk factors for post-TIPS OHE but the most important risk factors specifically studied, or specifically pertaining to TIPS insertion, are outlined here (Figure 3).

### 6.1. Demographic Risk Factors

**Age:** Older age has repeatedly been shown to be a predictor for post-TIPS HE, and particularly at ages >70 years the incidence rises considerably [54]. The liver maintains functionality at high ages, so the risk likely rests in changes in the aging brain, making it less resistant to neurotoxins and inflammation. Age-related sarcopenia with less ammonia-clearing capacity contributes [17,19,55,56,57,58]. A meta-analysis indicated an age threshold: age >70 but not >65 was associated with an increased post-TIPS HE incidence [54]. Still, high age alone should not exclude patients from TIPS, but particular attention to markers of other organ functions is necessary, e.g., sodium and creatinine [59].

There is limited evidence to suggest gender differences but the available literature suggests that males might have a higher risk than females [60]. 

### 6.2. Cirrhosis-Related Risk Factors

**Liver function:** Several studies confirm that a decline in pre-TIPS metabolic liver function is closely associated with the risk of developing post-TIPS HE [17,57,58,61,62,63]. However, it is not settled which liver function measures are critical. As to the liver disease stage, no suggested MELD threshold exists to contraindicate TIPS [59]. Child-Pugh class C is a relative contraindication as this score includes HE, cf. below.

**Pre-TIPS HE:** A history of overt HE (OHE) is a well-established predictor of post-TIPS HE [35,55,58], but carefully controlling pre-TIPS HE seems to mitigate that risk [57]. MHE grade I HE has also been reported to entail a higher risk of post-TIPS OHE, although evidence is scarce. In a study including 82 patients, an abnormal pre-TIPS portosystemic encephalopathy test (PSE) identified 77% of patients who developed post-TIPS HE, and the negative predictive value of a normal test was 80% [64]. In line with that, Berlioux et al. used the Critical Flicker Frequency test (CFF) to identify MHE before and several times after TIPS placement and found that OHE developed more often in patients with MHE diagnosed by CFF despite the absence of prior OHE; and that a normal CFF value equal to or greater than 39 Hz had a 100% negative predictive value for post-TIPS OHE [34]. A contradictory study using the PSE, CFF, and Animal Naming Test (ANT) found that HE risk increased with the number of abnormal tests while no single test was the most important [65]. Lastly, a Danish single-centre study where MHE was systematically diagnosed pre-TIPS using continuous reaction times (CRT) test found the overall OHE incidence was 38% during the first year post-TIPS, but when patients with MHE before TIPS were excluded, the incidence only decreased to 33% [28]. However, MHE detected during the first month after TIPS is reported to be associated with more than a three-fold increase in OHE incidence later on, suggesting that psychometric testing can be used for early post-TIPS HE risk evaluation [28]. Taken together, the prevailing data illustrate that MHE screening prior to, and in the weeks after, TIPS may be useful in detecting high-risk individuals, but larger studies are warranted with stratification according to OHE history, and systematic MHE testing is currently not the standard in most TIPS centres. 

Highly recurrent and persistent HE remains an absolute contraindication to TIPS unless TIPS is a rescue procedure in situations with planned liver transplantation [59]. 

**Hyponatremia:** There is a close relationship between plasma sodium levels and HE. At similar ammonia levels, the dominant EEG frequency was shown to be lower (worse) with lower serum sodium [50]. A more extensive study by Bossen et al. has since confirmed this association [66]. We found only one study that focuses specifically on hyponatremia as a risk factor for post-TIPS HE, and with pre-TIPS Na < 125 mEq/L, 125–129.9 mEq/L, 130–134.9 mEq/L, and ≥135 mEq/L the incidence of post-TIPS HE within one week was 37.5%, 25%, 25%, and 3.4%, respectively [61]. Thus, hyponatremia is likely a strong risk factor for post-TIPS HE.

**Pre-TIPS spontaneous portosystemic shunts (SPSS):** SPSS are present in at least 20% of cirrhosis patients, increasing the risk of HE after TIPS [67]. The most extensive study (*n* = 903) on the topic is a 2018 retrospective study from a Chinese military hospital, which found that patients with SPSS with a total diameter ≥6 mm had the highest post-TIPS HE risk, and shunt embolisation lowered the risk [67]. A similar result was observed in an earlier study [68]. These observational studies were backed up in 2022 by a trial of 56 patients with large SPSS, prospectively randomised to TIPS or TIPS + embolisation of SPSS, demonstrating that the latter group had fewer HE cases during the first year after TIPS (22% vs. 51%) [69]. Similarly, a retrospective study found that large paraumbilical vein shunts, as defined by cross-sectional areas > 83 square millimeters, diameter ≥ 8 mm, or greater than half of the diameter of the main portal vein, yielded a doubled 2-year post-TIPS HE risk (52% vs. 26%) [70]. Importantly, neither study found an increased risk of death, variceal rebleeding, or TIPS dysfunction after SPSS embolisation. Conversely, a smaller retrospective study of 33 patients found that embolisation of SPSS prior to TIPS did not lower the post-TIPS HE incidence compared to a control group without SPSS [71]. Taken together, the evidence suggests that SPPS should be sought out prior to TIPS, and the embolisation of large SPSS should be considered. Still, the issue is controversial because it is difficult to get a pathophysiologic grip on how the HE risk function from a spontaneous shunt differs from that of an iatrogenic shunt.

**Nutritional status:** Undernutrition is a very frequent challenge in cirrhosis patients and can contribute towards sarcopenia, [72]. Several studies confirm the relationship between poor body composition status and post-TIPS HE. Two studies found a thin or low-density psoas muscle CT scan to be associated with high post-TIPS HE incidence [33,73]. Other studies used skeletal muscle index (ratio of the muscle in arms and legs to height) as a measure of sarcopenia and found that 50 out of 56 patients with sarcopenia and none of the 6 patients without sarcopenia developed post-TIPS HE and that patients with improvement in skeletal muscle index reversing from sarcopenic to non-sarcopenic had a lower risk of developing post-TIPS OHE 6 months after TIPS [32,74,75]. However, not all studies show a significant correlation, and the predictive role of nutritional status and sarcopenia concerning the development of post-TIPS HE needs further investigation [76,77]. In other studies, adiposity is used as a marker of nutritional status. Here, male patients with a low volume of visceral fat and female patients with a low volume of subcutaneous fat pre-TIPS had a higher risk of post-TIPS HE, and patients that increased their body fat fraction after TIPS had a lower risk of developing post-TIPS HE [75,78].

### 6.3. Comorbidity-Related Risk Factors

**Type 2 diabetes and obesity:** Diabetes is a risk factor for post-TIPS HE [21,79,80,81]. Large retrospective studies report that oral treatment (not insulin) for diabetes is associated with a doubled risk (OR 1.9) [79,81]. The risk is probably rooted in microvascular changes in the brain and neuronal damage causing gut dysmotility, constipation, and bacterial overgrowth—all well-known diabetes complications that also increase the HE risk in cirrhosis in general [82]. Pre-TIPS severe obesity (BMI > 32) has also been suggested to be related to a higher incidence of post-TIPS HE [83]. More studies evaluating HE risk in patients on modern antidiabetics are needed, given the increase in obesity rates. 

**Renal function:** TIPS may be a treatment for hepatorenal syndrome, but on the other hand, impaired renal function is a risk factor for post-TIPS HE. Renal impairment is present in sicker, more decompensated patients, and the kidneys play an important role in ammonia clearance from the blood [84]. A small study including seventeen patients evaluated the impact of chronic kidney disease, i.e., not hepato-renal syndrome, on the risk of developing post-TIPS HE and found an incidence of 47% of new or worsening HE post-TIPS [85]. Likewise, another study found that Glomerular Filtration Rate (GFR) < 30 mL/min/1.73 sqm, haemodialysis, and chronic kidney disease at the time of TIPS placement were strong predictors of post-TIPS HE within 60 days, while GFRs between 30 and 60 and 60 and 90 were not negative predictors [86]. Other studies, not focusing specifically on chronic kidney disease, report that high creatinine is linked to a higher risk of post-TIPS HE [34,86]. 

**Proton-pump inhibitor use:** The use of proton-pump inhibitors (PPIs) in cirrhosis patients is associated with an increased risk of HE [87]. PPI use is also a risk factor for the development of post-TIPS HE; patients on PPI treatment at the time of TIPS insertion had a nearly tripled rate of post-TIPS HE (*n* = 397, 30.4% vs. 11.7%, *p* < 0.001). The post-TIPS HE rate increased with the PPI dose [88]. This finding is confirmed by a later study where PPI users have a doubled risk of post-TIPS HE 2.0 (*n* = 86) [89].

**Other medications:** Excess diuretics and ensuing dehydration are recognised precipitating factors for HE before and after TIPS. The roles of benzodiazepines, opioids, and other psychoactive medications as HE precipitants are uncertain, but the drugs are considered responsible for drug-related encephalopathy, which can add to HE [45]. However, no study has specifically examined the effect of these medications on HE risk in a pre/post-TIPS setting. 

### 6.4. Procedural Risk Factors

**TIPS timing:** A pre-emptive TIPS (p-TIPS) is now recommended in Child-Pugh class C and B cirrhosis patients (score 7–14) with variceal bleeding and active bleeding at index endoscopy despite Terlipressin treatment or high hepatic venous pressure gradient (HVPG) (a measure of portal pressure) > 20 mmHg at the time of bleeding [90]. This timing of TIPS placement has called for new evaluations of the HE risk. The question was recently approached using multicentre data from 671 European patients [91], and a meta-analysis including 1372 patients [92]. Both studies found that the post-TIPS HE rate was not higher than in the standard-of-care (SOC) groups (p-TIPS 38.2% vs. SOC 38.7% and p-TIPS 38% vs. SOC 35%). These findings are corroborated by a similar Chinese study in 262 patients [93]. 

**TIPS indication:** The TIPS indication impacts the risk of post-TIPS HE. After TIPS on the indication of acute uncontrollable variceal bleeding, the risk for OHE is up to 60% [94,95]. Considering the high mortality in this scenario, this risk may be considered acceptable given that most cases can be controlled, and prior HE in this setting is not a contraindication. Elective p-TIPS for variceal bleeding does not incur an increased HE risk compared to standard-of-care (≈35%) [91,92]. In this setting, refractory ascites and prevention of variceal bleeding are the most common indications, and here, a more careful selection of TIPS candidates is justified, often involving the exclusion of patients with high age and prior recurrent and persistent HE. Likewise, the elective scenario allows time for nutritional improvements and HE prophylaxis. Post-TIPS HE rates are, therefore, lower. A meta-analysis by Salerno et al. from 2007, including three randomised controlled trials of TIPS for refractory ascites found that the one-year cumulative HE risk of an HE episode was 20–30%, which was similar between the TIPS and paracentesis groups [96]. However, patients in the TIPS group had recurrent HE episodes more often. 

**Hepatic venous pressure gradient (HVPG) and portal pressure gradient (PPG) reduction after TIPS placement:** HVPG reduction of >9–10 mmHg or >60% by TIPS has been reported to increase the risk of post-TIPS HE [22,62]. However, a Swedish retrospective study of 131 TIPS patients found no difference in post-TIPS HE in groups with pressure gradients over or under five mmHg, but not all the patients had cirrhosis [97]. A recent study from 2023 suggests that individualised stent diameters may be warranted (cf. below): The authors included data from 2100 TIPS patients and observed that in cirrhosis patients in Child-Pugh A class, none of the PPG thresholds were discriminative of clinical outcomes, including HE. However, in Child-Pugh B class patients, a PPG of 12 mmHg showed a higher net benefit on other portal hypertension complications; in Child-Pugh C class patients, this was true for PPG < 14 mmHg [98]. 

**Stent diameter:** In line with the findings on HVPG and PPG reductions, the TIPS shunt diameter matters to the post-TIPS HE risk. Stent diameters of 8 mm and smaller are associated with lower post-TIPS HE, around 7–10% [27,35,99,100], while a diameter of >10 mm is an independent risk factor and carries a post-TIPS HE risk of up to 50% [62,101]. The connection between stent diameter and HE was demonstrated by Schepis et al., who conducted a study where stent under-dilatation to 6–7 mm was associated with a lower HE risk from 54% to 27% during the first year post-TIPS [99]. As stent diameter decreases, so does the intended hemodynamic improvement by the TIPS, and stent diameter choice should, therefore, always be carefully weighed against the individual risk factors, including TIPS indication and HE risk [102].

**Portal flow pattern:** A large study (*n* = 252) examined the importance of pre-TIPS portal blood distribution using portography using a portal venous branch puncture during the TIPS procedure. The study found that portal flow diversion influenced the risk of post-TIPS HE. In patients where the right portal vein received blood from the superior mesenteric vein instead of from the splenic vein or both, post-TIPS ammonia increased, and HE risk was higher (adjusted HR 3.70). Only 6% of patients had that particular portal flow pattern [103]. Another aspect of portal flow patterns is that up to 25% of patients with liver cirrhosis with time may develop hepatofugal portal flow, that is, a reversed or alternating portal flow direction, which entails a worse overall prognosis compared to those maintaining normal hepatopetal flow. However, these patients are largely protected against post-TIPS HE because the stent, while normalising their portal flow direction and pressure, does not change the fraction of portal blood bypassing the first passage through the functional liver. They thus may be candidates for TIPS despite their advanced disease stage [104,105,106].

## 7. Predictive Models and Methods

Predictive models based on a combination of risk factors have been developed for predicting post-TIPS HE [25,107,108]. Some of the models include a high number of supposed predictor variables; they all need further validation, and none have gained widespread clinical recognition. One study aimed to determine if the Freiburg Index (bilirubin, creatinine, age, and albumin) could predict post-TIPS HE, and found an area under the curve (AUC) of 0.74 indicating a good discriminatory ability of the index [109]. The Freiburg Index was initially developed in 2021 to estimate post-TIPS survival with good discriminative ability in the original German cohort but with less impressive results in the Chinese and Danish cohorts [13,88,110]. Similarly, another study combined clinical and laboratory markers (age, Child-Pugh Score, diabetes, serum creatinine, and sodium) and devised a predictive score with an AUC of 0.81 [79]. A recent study expanded the variables and included imaging characteristics as well as clinical findings. The scoring system consisted of bilirubin level, Child-Pugh score, hepatic fissure maximum width, and diameter ratio of the portal versus splenic vein, and found an AUROC of 0.97 [107]. The albumin-bilirubin (ALBI) score, developed initially to estimate survival in hepatocellular carcinoma (ALBI = (log10 bilirubin × 0.66) + (albumin × −0.085), where bilirubin is in μmol/L and albumin in g/L), has been evaluated for post-TIPS HE prediction with an AUC of 0.74 [111]. 

Finally, an Italian study examined whether an induced ammonia challenge could help identify TIPS recipients who will develop HE [112]. Unexpectedly, patients with lower ammonia levels prior to the challenge were at greater risk of developing HE post-TIPS during a 12-month follow-up. This finding may be related to patient selection, where all were considered safe TIPS candidates with no HE history and an evident ability to tolerate some hyperammonaemia.

## 8. Upcoming Predictive Markers

New imaging and biomedical technologies are being deployed to search for better prediction markers and models. These include CT-based radiomics [113], and various circulating markers identified by metabolomic and proteomic strategies [29,114,115,116]. It has been suggested that a decreased post-TIPS concentration of three specific conjugated di- and tri-hydroxylated bile acids and glycerophosphocholine is correlated with post-TIPS HE grade [115,116]. Both substances act directly in the brain, suggesting that post-TIPS HE risk may be related to cognitive effects of liver exocrine patterns [117]. Also, low serum cholinesterase, which is almost exclusively synthesised in hepatocytes and, in this context, may be seen as a proxy measure of liver function, was associated with post-TIPS HE risk in a large German cohort study [118]. 

## 9. HE Prophylaxis

### 9.1. Pharmacologic Prophylaxis

The cornerstones of pharmacological prophylaxis of HE are lactulose, rifaximin, or a combination of both. Still, only four studies (three within the past ten years, Table 1) have examined the effect of these treatments for prophylaxis of post-TIPS HE. The first study from 2005 by Riggio et al. thoroughly matched groups regarding central features but found no effect of lactitol (powder form lactulose) or rifaximin alone or in combination versus placebo on post-TIPS OHE [119]. In 2021, however, Seifert et al. demonstrated that lactulose and rifaximin in combination could reduce post-TIPS HE, but only in patients with a history of OHE [55]. Also, in the French multicentre study of high-risk TIPS candidates by Bureau et al. from 2021, rifaximin administered 14 days prior to TIPS and six months after was shown to reduce the absolute risk of HE by 20% compared to placebo in patients with primarily alcohol-related liver cirrhosis while the HE incidence was still high [120]. Beyond lactulose and rifaximin, one Chinese study excluding patients with prior OHE examined the effect of L-Ornithine L-Aspartate (LOLA) on OHE in a randomised trial of 133 patients and found no effect [121].

Reviews and meta-analyses have scrutinised the data from the treatment studies. The two most recent studies found that neither lactulose alone, rifaximin alone, nor a combination of both significantly reduced the incidence of post-TIPS HE [122,123]. Furthermore, they emphasize that the evidence is scarce, and more studies on combination therapy are needed. So, despite a strong notion that HE prophylaxis is beneficial in high-risk patients, it remains unclear if HE prophylaxis is merited and, if so, in which patients. A much-anticipated European multicentre randomised controlled trial is currently examining the prophylactic effects of lactulose and rifaximin on post-TIPS HE, and data collection is expected to be complete by the end of 2023 [124]. 

### 9.2. Procedural Preventive Measures

The risk of post-TIPS HE is higher in patients with large SPSS, and the available evidence suggests that shunt embolisation can reduce this risk without increasing the risk of complications (cf. above) [67,68,69,70,71]. Accordingly, quantifying SPSS prior to TIPS and considering embolisation is increasingly being applied in clinical practice. Also, during TIPS procedures, the absolute and percentwise reduction of PPG should be monitored and kept below 60% as larger pressure gradient drops increase the risk of post-TIPS HE [22,62]. It has been suggested that in Child-Pugh class B cirrhosis patients, a PPG of 12 mmHg might have the best net benefit, while in Child-Pugh C patients, this was true for PPG < 14 mmHg [97]. In line with this and as mentioned previously, the stent diameter is of importance, and Child-Pugh B and C patients may have difficulties tolerating stent diameters > 8 mm, while patients with less severe disease may tolerate wider stents [27,35,62,101,102]. The goal seems to choose the lowest possible stent diameter effective for the clinical problem at hand. 

## 10. Treatment of Post-TIPS HE

### 10.1. Pharmacologic Treatment Options

Post-TIPS HE is treated like HE in general with lactulose and, if necessary, also rifaximin. Precipitating factors beyond the newly placed TIPS should be sought for and managed, and nutritional therapy should be part of the standard of care. However, approximately six percent of patients have frequently recurrent or persistent post-TIPS HE despite lactulose and rifaximin treatment (cf. above). Second-line pharmacological options should be considered in these cases, including branched-chain amino acids (BCAA), LOLA, and newer drugs aiming to disrupt ammonia formation [125]. Gut microbiota composition, e.g., with urease active bacteria, might be related to the occurrence and severity of post-TIPS HE, and gut microbial screening and faecal microbiota transplant (FMT) might prove a treatment option in the near future [126]. A larger European trial is currently studying the effects of albumin treatment on outcomes in liver cirrhosis and will also provide new data on post-TIPS effects [127].

### 10.2. Procedural Treatment Options

Secondary reduction in the stent diameter has been reported to improve HE in some patients with worsening or occurrence of post-TIPS HE without increasing the risk of new variceal bleeding, but often with partial recurrence of ascites [35,36,64,128,129,130,131]. The studies on the topic report improvement in HE in 80–90% of patients after TIPS reduction, but survival, at least in one study, was only 10% at one year without liver transplantation [35,129]. A single study, however, reported a somewhat lower response rate of 55% [131]. The effect of reduction of stent diameter on HE remains uncertain and is usually only considered for cases of persistent or recurrent HE despite pharmacologic treatment and is not first-line therapy [132]. The complete closure of the stent is also an option [133]. The ultimate treatment for post-TIPS HE is a liver transplant in eligible patients 

## 11. Conclusions

Post-TIPS HE is the Achilles heel of this otherwise efficacious splanchnic hemodynamic intervention for portal hypertension and its serious complications. Several well-designed studies report on the issue, even in studies that do not have HE as their primary endpoint. The heterogeneity among cohorts and reporting strategies hinders clear generalisation. Still, despite targeted patient and procedure selection and attempts at pharmacological risk reduction, the prevailing message is that the post-TIPS HE incidence has remained constant during the past decade (Figure 2). Given the modifiable nature of several risk factors, many HE-related readmissions may be preventable in the same way as in liver cirrhosis without TIPS. The available evidence suggests that the following measures would be reasonable to take to prevent post-TIPS HE:

- Pre-TIPS correction of sodium and nutritional status in patients with high HE risk undergoing elective TIPS.
- Stop PPI use unless strictly indicated.
- Consider closure of large SPSS.
- Optimize intraprocedural techniques, including attention to PPG reduction and using the smallest effective stent diameter.
- Early follow-up, including psychometric monitoring for early HE detection and patient education.

Moving forward, prospective rather than retrospective, case series implementing strict and systematic patient selection, HE prophylaxis, and small-diameter TIPS are needed to assess the effect of recent years’ practice. Also, as preemptive TIPS becomes the standard, comparisons to carefully matched patients without TIPS are required in order to inform on the added HE risk of receiving a TIPS. This risk is essential to substantiate because the newer studies and the meta-analyses of pre-emptive TIPS studies leave the notion that the extra risk incurred by TIPS may not be decisive [91,92]. If so, the management of post-TIPS HE should mostly be directed at pre-TIPS improvement in the clinical state of potential TIPS candidates, according to general recommendations for such patients, rather than in specific TIPS-related conditions. There is increasing attention to this need, and reviewing the existing newer literature leaves the impression that within the foreseeable future, we will have data to enter a more HE-safe TIPS era. 

## Figures and Tables

**Table 1:** Overview of the studies that have examined the effect of pharmacological prophylaxis of post-TIPS hepatic encephalopathy.

Studies on Pharmacological Prophylaxis of Post-TIPS Hepatic Encephalopathy						
Study	No. of Patients	Type of Patients	Indication (Bleeding/Ascites/Both)	Age (Mean, SD)	Medication (No. of Patients)	Effect
Riggio et al., 2005 [119]	75	25% alcohol cirrhosis Child-Pugh A/B/C (%): 27/56/17 Previous overt HE: 15%	50/25/0	57 (11)	Lactitol (25) vs. rifaximin (25) vs. SOC (25)	No effect of either lactitol or rifaximin on 1-month incidence of HE
Seifert et al., 2021 [55]	233	52% alcohol cirrhosis Child-Pugh A/B/C (%): 27/62/12 Previous HE: 21%	77/115/41	58 (19–80) (median, range)	Lactulose (85) vs. Rifaximin (6) vs. Lactulose + Rifaximin (59) vs. SOC (83)	Lactulose + Rifaximin prevented HE recurrence at 1, 3, and 12 months after TIPS, but did not prevent HE in patients with no history of HE
Bureau et al., 2021 [120]	197	86% alcohol cirrhosis Child-Pugh score: 8 (1) (mean, SD) Previous overt HE: 13%	37/160/0	60 (8)	Rifaximin (97) vs. placebo (100)	Rifaximin reduced the risk of overt HE within 168 days post-TIPS (odds ratio, 0.48 [CI, 0.27 to 0.87]).
Bai et al., 2014 [121]	40	75% hepatitis B virus cirrhosis Child-Pugh A/B/C (%): 45/18/38 No previous overt HE	36/4/0	48 (10)	I.v. L-ornithine-L-aspartate (LOLA) (21) vs. placebo (19)	LOLA reduced the increase in venous ammonia levels and improved psychometric scores for 7 days post-TIPS

## Data Availability

Not applicable.
